# Abstracts of the Annual meeting of the PCSI, 2008

**Published:** 2008

## Diagnostic discrepancies in pediatric echocardiography: An experience from a tertiary care centre in India

**Pankaj Bajpai, Sejal Shah, Harini C, PV Suresh, Sunita Maheshwari.** *Department of Pediatric Cardiology, Narayana Hrudayalaya, Bangalore.*

**OBJECTIVE:** To determine the incidence of discrepancies in the echo reports from peripheral centers compared to our center.

**METHODS:** One thousand consecutive patients with new registrations who came to pediatric echo room were studied. All echoes were performed by Consultant Pediatric Cardiologists on a GE Vivid-7 machine. Outside echo reports were reviewed separately. Subsequently, our reports were co-related with the cardiac CT, catheterization and surgical findings when available.

**RESULTS:** Outside echo reports were not available in 106 (10.6%) cases. Of the remaining 894 cases, significant discrepancies were observed in 127 (14.2%) cases. Mean age was 3.4 years with 67 (56.8%) cases being infants. Errors were noticed in relation to great vessels origin/malposition (n=22;17%), pulmonary venous return (n=22;17%), intra/extra-cardiac shunts (n=15;12%), semilunar valve disease (n=15;12%), coarctation of aorta (n=11;8.7%), coronary anomalies (n=6;4.7%), atrioventricular valve disease (n=6;4.7%) and presence of CHD in previously diagnosed rheumatic heart disease (n=3;2.4%). Aortopulmonary window (APW) was missed in 4 (3%) patients. Three patients referred as dilated cardiomyopathy turned out to have anomalous left coronary artery from pulmonary artery (n=2) and coarctation of aorta (n=1). Eighteen (14%) patients referred as ASD were found to have other diagnoses like APW,TAPVC etc. Ten (7.9%) patients with diagnosis of primary pulmonary arterial hypertension were found to have structural heart disease like VSD, TAPVC, APW etc. Five (3.9%) patients of CHD were labeled as normal previously. Outside Echocardiography was done by Adult Cardiologists, Pediatric Cardiologists and technicians in 118 (93%), 3 (2.3%) and 6 (4.7%) patients respectively. CONCLUSION: Significant errors in echo diagnosis of CHD affecting patient management were noted in 14% of patients referred from peripheral centers. Personnel doing pediatric echocardiography need specialized training in order to minimize misses. Training in systematic segmental approach and the use of tele-echo are potential ways of preventing these errors.

## Hemodynamic patterns in head-up tilt test in children with unexplained syncope

**Saji Philip, Leni Alexander, Anand Sreenivasan, Madhu Paulose, Saji Jose, Joy Thomas**^1^**, Kottureth Mammen Cherian**^1^**.** *Department of Cardiology, Division of Pediatric Cardiology, St Gregorios Cardio-Vascular Center, Parumala, Kerala, ^1^Division of Electrophysiology, International Center for Cardio Thoracic and vascular Diseases, Chennai, India.*

**AIM:** Unexplained syncope may cause diagnostic and therapeutic problems in children. The head-up tilt test (HUTT) has been shown to be a useful tool for investigating unexplained syncope. The prospective study, to explore on different hemodynamic patterns during the course of HUTT in children with unexplained syncope, and also to evaluate the feasibility of recommending tilt test in pediatric population to diagnose neural mediated syncope (NMS).

**METHODS:** 26 pediatric patients aged 5 to18-years, including 15 male and 11 female, with unexplained syncope with the mean course of 6 +/- 04 months, undergoing a standardized head-up tilt-table testing protocol evaluated by head-up tilt to 60 degrees for 20 minutes. The test was considered positive if syncope or presyncope developed in association with hypotension, bradycardia, or both. If tilting alone did not induce symptoms (syncope or presyncope), isoproterenol infusion was administered with increasing doses (0.02-0.08 *µ*g/kg per minute) to increase the heart rate 30% above the base line heart rate. According to their different hemodynamic patterns, they were divided into vasovagal response pattern, postural orthostatic tachycardia syndrome (POTS), orthostatic hypotension (OH) and normal response pattern. The vasovagal response was further divided into vasodepressor, cardioinhibitory and mixed patterns.

**RESULTS:** 19 of the 26 children with unexplained syncope displayed the hemodynamic pattern of vasovagal response (73%) and 7 showed HUTT negative response (27%). Among the positive cases, 18 (94.7%) were vasovagal type including 10 (52.6%) showed the pattern of mixed response, 06 (31.5%) vasodepressor response, and 2 (10.5%) cardio-inhibitory response. One patient (5%) showed POTS response. Among the normal response group, four cases were neuropsychiatric (n=1), conversion reaction (n=1), arrhythmias (n=2). Patterns of dysautonomic response and chronotropic incompetence were not observed in these children with unexplained syncope. The age of the children underwent HUT test was 12years +/-7 years. There was no significant difference in the baseline blood pressure among the children with vasovagal response, POTS and normal response. There were also no significant differences in the hemodynamic pattern to the age and sex ratio. No difficulties encountered during the test procedure and complications all were managed well.

**CONCLUSIONS:** The head-up tilt test is a noninvasive, sensitive, specific diagnostic tool and feasible to advise in children for evaluating children with unexplained syncope.

## Ebstein's anomaly – presentation, management options and outcome in the current era

**G Madhusudan, Suraj Varma, P Sreeja, J Vimala, Ulhas M Pandurangi, John Valliath, R Suresh Kumar.** *Institute of Cardiovascular Diseases, Madras Medical Mission, Chennai.*

**OBJECTIVE:** To critically evaluate the presentation and management options – medical, surgical, electrophysiologic – in Ebstein's anomaly across a wide spectrum of age and to assess the impact on outcome.

**MATERIALS AND METHODS:** Retrospective study of 43 consecutive cases of Ebstein's anomaly seen in a tertiary care center during January 2004 – June 2008.

**RESULTS:** Age 16 days – 52 years (median 9 years), Male:Female = 16:27. Presenting symptoms included none (18), exertional dyspnoea (13), paroxysmal palpitation (14) and syncope (6). 11 patients had SPO2 < 92%. 29 patients had cardiomegaly. ECG showed sinus rhythm (all), RBBB / polyphasic QRS complexes (17), and WPW syndrome (5). Echocardiography showed inferior displacement of septal leaflet (all), atrial shunt (24), ventricular shunt (6), RVOT obstruction (3), significant Tricuspid regurgitation (25), LTGA (2), and Situs inversus / dextrocardia (1). Management options: Group A (n=18) follow up, Group B (n=11) medications (antiarrhythmics 5, antifailure drugs 5), Group C (n=7) Electrophysiologic study and RF ablation and Group D (n= 7) surgery. All patients in group C showed accessory pathways (right posteroseptal 3, right free wall 1, right midseptal 1, left free wall 1, multiple 1). Inducible arrhythmia was found in 5. RF ablation was successful in 6. Indication for surgery (Tricuspid valve repair 2,tricuspid valve replacement 2, 11/2 ventricle repair 2, modified BT shunt 1) was severe TR with progressive cardiomegaly or severe hypoxia. There was no mortality. At a median follow up of 16 months all patients in Group A remained in functional class I/II, 1 patient in Group B underwent RFA but continued to have SVT on antiarrhythmics. One patient in Group C was on antiarrhythmics drugs. Operated patients were in functional class I/II.

**CONCLUSIONS:** Initially asymptomatic Ebstein's anomaly tends to stay so subsequently. Surgery/RF ablation in indicated cases could be performed with zero mortality and good functional outcome in the intermediate term.

## Cardiopulmonary exercise testing after surgical repair of tetralogy of fallot – A preliminary report

**G Madhusudan, P Atul, L Varatharajan, Jayanthi L, P Sreeja, J Vimala, R Suresh Kumar.** *Department of Pediatric Cardiology, ICVD, Madras Medical Mission, Chennai.*

**INTRODUCTION:** Cardiopulmonary exercise testing (CPT) is a powerful tool for objective evaluation of the cardiovascular, respiratory and muscular systems under controlled metabolic stress. Impaired CPT parameters could be a predictor for adverse events on long term follow-up.

**OBJECTIVE:** To assess cardiopulmonary exercise parameters after surgical repair of Tetralogy of Fallot (TOF) and to compare them with surgical Atrial Septal Defect (ASD) closure patients, and normal controls.

**METHODS:** In an ongoing study, 10 patients > 10 years (Group1, range 10 – 36 years, median 19.5 years) who had undergone Intra Cardiac Repair (ICR) for TOF at a median age 10 years, underwent CPT using upright bicycle ergometry with MedGraphics BreezeSuite PFX Ultima™, till exhaustion. Their exercise parameters [percentage of maximum oxygen consumption (VO2max) of the predicted, oxygen consumption at anaerobic threshold (VO2AT)/VO2max, respiratory exchange ratio (RER), minute ventilation/carbon dioxide production (VE/VCO2) ratio and normalized maximal exercise performance (MEP) (Watt/kg)] were compared with a similar number of post operative ASD patients (Group 2), and normal controls (Group 3).

**Figure 1:**
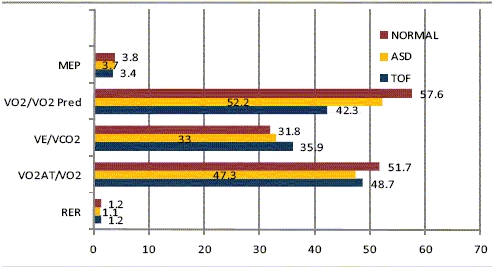
Comparison of exercise parameters

**RESULTS:** Comparison of exercise parameters [Figure 1].

**CONCLUSION:** The early results indicate uniformly impaired exercise parameters in surgically repaired TOF patients,even though the results were statistically significant only for VO2 max%. This impairment is probably disease specific and not an effect of sternotomy/ thoracotomy as post operative ASD patients behaved like controls. This hypothesis and its correlation with ECG and Echo abnormalities is being studied in a larger series for validation

## Extracorporeal membrane oxygenation as an evolving treatment modality for low cardiac output state in developing countries

**Singh Vishal, Priya Poonkodi, Sharma Rajesh, Varma Amit.** *Department of Pediatric Cardiac Surgery and Intensive Care Escort's Heart Institute and Research Centre, New Delhi.*

**BACKGROUND:** Veno-arterial extracorporeal membrane oxygenation (ECMO) is a common modality of circulatory assist device used in children. We assessed the outcome of children who had rescue ECMO following repair of congenital cardiac defects (CCD) in our hospital.

**METHODS:** From September 07 to September 08, 13cases underwent veno arterial extracorporeal membrane oxygenation (ECMO) and all the cases had intra thoracic cannulation. Of these 8 had undergone arterial switch operation (ASO) for D-Transposition of great arteries (D-TGA) with severe left ventricular (LV) dysfunction, 5 cases had elective ECMO post cardiac surgery due to regressed left ventricle. 2 cases required emergency ECMO following cardiac arrest in the intensive care unit (ICU) and one case required ECMO for acute respiratory distress syndrome (ARDS) due to gram negative sepsis. Three cases required ECMO following prolonged cardiac arrest after intracardiac repair for Tetralogy of Fallot and Rastelli procedure for tetralogy of fallot and pulmonary atresia. Two cases required emergency ECMO following prolonged cardiac arrest after severe pulmonary artery hypertensive (PAH) crisis with severe right ventricular (RV) dysfunction.

**RESULTS:** The mean age and weight were 184.45±181.69 days and 6.79±3.91 kg respectively. ECMO was successfully weaned off in 69.2 % ( 9/13) and the mean duration of ECMO was 97 hours. One case required premature weaning off ECMO due to excessive bleeding. Eight cases were successfully discharged and the mean duration of ICU stay was 22 days after weaning off ECMO. Four patients, three with RV dysfunction following TOF repair and TAPVC repair and one with ARDS due to gram negative sepsis could not be weaned off ECMO and expired. One patient had ventricular dysrhythmia on ECMO and three cases had choreoathetoid movements following ECMO weaning which responded very well to oral sodium valproate.

**CONCLUSION:** ECMO has a promising role as a temporary bridge in patients who need support both for acute cardiac or respiratory dysfunction.

## Clinical impact of vasopressin infusion in cardiac arrest and refractory hypotension in the pediatric cardiac ICU

**Agrawal Amit, Singh Vishal, Sharma Rajesh, Varma Amit.** *Department of Pediatric Cardiac Surgery and Intensive Care Escorts Heart Institute and Research Centre, New Delhi.*

**AIM:** To assess the effect of vasopressin on hemodynamics when used as an infusion for refractory hypotension and post cardiac arrest.

**MATERIAL AND METHODS:** A prospective study conducted over a duration of 6 months from February 08 to august 08 in a tertiary pediatric cardiac critical care unit. Twenty patients (median age 4.5 months ) who underwent surgical correction for a varied spectrum of complex congenital heart disease and who required vasopressin infusion for more than 60 mins were included. Vasopressin infusion was started following cardiac arrest in 9 patients & in 11 cases was instituted for refractory hypotension resistant to routine catecholamine infusion. Vasopressin was administered as a bolus during cardiac arrest at 0.4 units /kg/dose, if the patient was unresponsive to multiple adrenaline boluses and the infusion was administered within the dose range 0.0001 to 0.003 units/kg/min.

**RESULTS:** The mean arterial blood pressure (ABP) improved from 42±15.3 mmHg to 58±16.8mmHg after one hour and after 24 hours the mean ABP improved to 68±19.3 mmHg. There was no significant increase in the heart rate following vasopressin infusion. Also in 9 cases the inotropes were successfully tapered & inotrope score was decreased from 23.97 to 20.82 within 24 hours of AVP infusion. There was no significant alteration observed in urine output, serum sodium, and platelet count. The median duration of vasopressin infusion was 55.5 hrs (12hrs to 237 hrs). No significant adverse effects involving the digital and splanchnic circulation or renal or hepatic function were observed. Two cases expired while on vasopressin infusion, due to severe low cardiac output state and three cases due to drug resistant gram negative sepsis eventually.

**CONCLUSION:** Vasopressin infusion does have a role in improving the hemodynamics in cases with advanced shock and cardiac arrest, especially in catecholamine resistant shock.

## Cardiac functions in children with HIV infection

**Loncy V, Nidhi Narula, Surjit Singh, Rohit Manoj Kumar.** *Department of Cardiology and Pediatrics, PGIMER, Chandigarh.*

**BACKGROUND:** Infants and children with HIV infection are a rapidly expanding population.As compared with adults, there is paucity of literature regarding cardiovascular manifestations of HIV infection in children.

**OBJECTIVE:** To know the pattern of cardiac involvement in an Indian set up and to correlate the degree of cardiac dysfunctions and complication with the degree of immune suppression as assessed by CD4 counts.

**METHODS:** Between Jan 2006 to June 2007, 40 Children with diagnosed HIV infection were prospectively studied. They were divided into 2 groups, Gr I-Asymptomatic not on ART, Gr II –symptomatic children on ART. All of them underwent physical examination, CXR, ECG and detailed Echocardiography. Hemogram and CD4 count also recorded.

**RESULTS:** Mean age was 7.1 ± 2.6 years, 70% had weight for age and height for age <3^rd^ centile. Children on ART tended to have higher resting heart rate. All had normal sinus rhythm. 35% of children in Gr II had cardiomegaly. There were no statistically significant differences in the LVed/LVes dimensions, LVEF, FS and LV mass between the 2 groups. Average LVEF was 65.05 ± 6.54%. One patient in Gr II had LV systolic dysfunction with LVEF of 41% and FS -19%. 15% (n=3/20) of patients in Gr II had PAH in contrast to none in Gr I. None had pericardial effusion. The average CD4 count for all children was 649.5± 318.06/mm3.Gr II children had a statistically significant (p=.02) lower CD4 count as compared with Gr I children. Lowest CD4 count was 74/mm3 seen in a Gr II child with LV systolic dysfunction.

**CONCLUSION:** Cardiac problems do occur in children with HIV infection, with PAH being increasingly seen in these children. Physician caring for children with HIV infection should have a high index of suspicion.

## Echocardiographic and electrocardiographic evaluation in multi-transfused patients with β thalassaemia major: can tissue doppler imaging predict early cardiac dysfunction?

**Nidhi Narula, RK Marwaha, Rohit Manoj Kumar.** *Department of Cardiology and Pediatrics, PGIMER, Chandigarh.*

**BACKGROUND:** Life expectancy in patients with thalassaemia major is still limited by development of congestive heart failure due to a cardiomyopathy, associated with iron over-load. Aggressive chelation therapy may prevent, delay or even reverse myocardial dysfunction, but once overt heart failure develops, only 50% of patients survive. This study assesses early myocardial involvement in thalassemic patients with no clinical evidence of cardiac dysfunction.

**METHODS:** This is an interim study conducted on 70 patients admitted in the thalassaemia ward, Advance Pediatric Centre, PGIMER for transfusion and chelation therapy from February 2007 to Aug 2008. Patients divided into two groups. Gr I (n=35)-ß thalassemic patients who had received multiple transfusions, not on chelation therapy. Gr II (n=35)-ß thalassemic patients who had received multiple transfusions, on chelation therapy. All patients underwent clinical examination, detailed echocardiographic evaluation-M-mode, 2D Doppler and Tissue Doppler Imaging (TDI), with focus on assessment of ventricular systolic function and diastolic function, which included E/A, IVRT and Index of Myocardial Performance (IMP),ECG and Holter 6 monthly.

**RESULTS:** Mean age-17 years (range 5-30 yrs), Mean weight- 38 kgs (range 14 – 67 kgs).RV diastolic dysfunction was seen in 25% (n=17/70) patients,7 (20%) in Gr I and 10 (29%) in Gr II. RV systolic dysfunction in 3% (n=2),one patient in each group.4 patients (5.7%) had LV systolic dysfunction, 50% in each group.2 patients in Gr II had LV diastolic dysfunction. One patient had rhythm abnormality (first degree heart block with prolonged QT interval) in Gr I. TDI could detect diastolic dysfunction in 2 patients, not detected by 2D Doppler.

**CONCLUSION:** Cardiac involvement is characterized by primary diastolic and late systolic dysfunction with a common right ventricular impairment. This is a limited study, still needs to see the effect of chelation therapy.

## Profile of congenital heart disease in patients with 22q11 microdeletion

**Mukesh Kumar Singh, Sejal Shah, Meenakshi Bhat**^1^**, Sunita Maheshwari.** *Departments of Pediatric Cardiology and ^1^Clinical Genetics, Narayana Hrudayalaya Institute of Cardiac Sciences, Bangalore.*

**INTRODUCTION:** 22q11microdeletion is the commonest microdeletion syndrome in humans, and is commonly associated with congenital heart disease (CHD). Our study aimed to study the types of CHD in patients with the 22q11 microdeletion

**MATERIAL AND METHODS:** Syndromic patients with conotruncal anomalies, and patients with typical facial dysmorphism were screened by Fluorescent In-Situ Hybridization (FISH). All had detailed transthoracic echocardiography. Thirteen underwent cardiac computerized tomography (CT).

**RESULTS:** We identified 53 patients with 22q11 microdeletion by FISH. One patient had a normal echocardiogram; 4 had lesions not significant clinically; 48 (90%) had important CHD including Pulmonary Atresia–Ventricular Septal Defect (PA-VSD) (n=18,34%), Tetralogy of Fallot (TOF) (n=14,26%), Truncus Arteriosus (n=7,13%), Atrial Septal Defect (n=4, 7%), Ventricular Septal Defect (VSD) (n=3,5.5%) and others (n=2,3.5%). Forty-four (83%) patients had VSD as part of their CHD, 42 of these being malaligned. A right aortic arch, including one cervical arch, was found in 14 (26%) patients, and an aberrant subclavian artery in 5 (9.5%). In this series, there was no patient with interrupted aortic arch.

In the pa-vsd group, long-segment atresia was present in 16 and membraneous atresia in 2. Confluence of central pulmonary arteries could not be demonstrated in 4 patients, even on ct. 14 Patients had large (>3mm) major aortopulmonary collateral arteries (mapcas). In the truncus group, 4 patients had type i truncus, 2 had type ii truncus and the left pulmonary artery was absent in one.

**CONCLUSIONS:** This is likely the largest single center Indian series of patients with FISH-proven 22q11 microdeletion. This syndrome has a variable cardiac phenotype that ranges from normal to significant CHD wherein PA-VSD and TOF predominate.

## Clinical and hemodynamic predictors of left ventricular systolic dysfunction after percutaneous closure of patent ductus arteriosus

**Rama Krishna SVK, Krishnamoorthy KM, SS Padhi, S Sivasankaran, Ajith Kumar VK, Thomas Titus, Tharakan JM.** *Department of Cardiology, Sree Chitra Tirunal Institute for Medical Sciences and Technology, Trivandrum, Kerala, India.*

**AIM:** To analyse the clinical and hemodynamic predictors of Left Ventricular (LV) systolic dysfunction after percutaneous closure of Patent Ductus Arteriosus (PDA).

**MATERIAL AND METHODS:** Patients who underwent percutaneous PDA closure were analysed retrospectively. Those who had an LV ejection fraction (LVEF) of <50% or a fall in LVEF of ≥ 10% after PDA closure were defined to have LV systolic dysfunction. Controls were selected as age matched subjects who underwent PDA closure but who didn't satisfy these criteria. The basal characteristics, echocardiographic parameters and catheterisation data were analysed.

**RESULTS:** Fifty subjects with LV dysfunction (group A) and 31 controls (group B) were identified. Group A had higher baseline LV end diastolic diameter (LVIDD 50.52 ± 13.68 vs. 43.97 ± 10.15; p = 0.024), end systolic diameter (LVIDS 32.64 ± 10.87 vs. 26.84 ± 6.43; p=0.009) and lower LVEF (66.56 ± 7.21 vs. 70.03 ± 6.77; p =0.034). The mean z scores of LVIDD and LVIDS were also higher (3.45 ± 1.54 vs. 2.42 ± 1.61; p = 0.005 and 2.69± 1.55 vs. 1.87± 1.06; p = 0.001 respectively). Patients in group A had a significant fall in LVIDD after the procedure but not in LVIDS (p = 0.001 and 0.19 respectively) whereas group B had a fall in both parameters. There was no significant difference in the angiographic PDA size or hemodynamic parameters. The post procedure LVEF correlated best with basal LV dimensions which in turn correlated with the size of the PDA. A cut off of 2.5 for the z score of basal LVIDD predicted LV dysfunction with a sensitivity of 62% and specificity of 71%.

**CONCLUSION:** Larger PDA results in adverse LV remodelling which may lead to LV systolic dysfunction after closure. The basal LV function parameters are the best predictors of post procedural LV dysfunction.

## Vascular function in patients after coarctation repair and relevance of age at surgery

**Sanmugasundaram, SS Padhi, Krishnamoorthy KM, S Sivasankaran, Ajith Kumar VK, Thomas Titus, Tharakan JM.** *Department of Cardiology, Sree Chitra Tirunal Institute for Medical Sciences and Technology, Trivandrum, Kerala, India.*

**AIM:** To assess conduit artery function in upper limb in patients who underwent successful coarctation repair at various ages

**METHODS AND RESULTS:** Flow-mediated dilatation (FMD) and the dilatation after sublingual nitroglycerin were measured by using high-resolution ultrasound in the brachial artery in 24 coarctation patients (42% males and 58% females, aged 17±10 years; median age at operation) and 22 control subjects (59 % males and 41 % females, aged 18±10 years). Patients, compared with control subjects, had lower brachial FMD (7.72±2.7% versus 10.38±3.51%, respectively; P=0.008) and NTG (12.1±2.4% versus 14.6±4.2%, respectively; P=0.02). Age at the time of repair was related to brachial FMD & NTG (p=0.05 & 0.005 respectively).

**CONCLUSIONS:** Patients with repaired aortic coarctation have impaired conduit artery function, with abnormal responses to flow and NTG, in the upper part of the body. Early repair is associated with preserved reactivity of conduit arteries.

## Bidirectional cavopulmonary shunt in infancy – short term outcome

**Prem Alva, Roy Varghese, N Poornima, Sunnykutty Zacharias, John Valliath, R Suresh Kumar.** *Department of Pediatric Cardiology and Pediatric Cardiac Surgery Institute of Cardiovascular Diseases, Madras Medical Mission, Chennai.*

**INTRODUCTION:** The bidirectional cavo pulmonary shunt, is usually the first stage procedure for hearts with single ventricle physiology. This report studied the short term outcome following the procedure performed in infancy as compared to that performed later in life.

**MATERIALS AND METHODS:** Between January 2003 and September 2008, 75 patients underwent the bidirectional cavo pulmonary shunt at our unit. Of these 28 (37%) were performed in infancy (Group I); mean age 7.8 months. Group II comprised 47 patients. Group II patients were further divided into II (a)- one to five years, II (b)-five to ten years and II (c)- more than ten years. Mean SpO_2_ was 71.6% on room air in Group I, while the same in Group II was 80.4%. 23 patients underwent the pulsatile bidirectional cavo pulmonary operation of which 8 (28%) belonged to Group I. Variables assessed were anthropometric data, superior caval pressures, duration of ventilation, ICU stay and hospital stay and morbidity. Patients were followed up at intervals of three, six and twelve months.

**RESULTS:** Outcome variables in the two groups and the three sub groups were assessed and statistically compared. There was no hospital mortality. The study showed no significant differences in the immediate and short-term outcomes following the procedure in either group. Oxygen saturations were higher at three months (85.4%) and at one year (79.2%) in those who underwent the procedure in infancy as compared to the pre operative levels.

**CONCLUSION:** The bidirectional cavo pulmonary shunt can be safely performed in infancy with significant improvement in the systemic oxygen saturation. This could translate into improved quality of life and growth without adversely affecting the immediate hospital course.

## Early onset pulmonary hypertension in atrial septal defects-association with a left superior vena cava to coronary sinus

**Sripadh Upadhya, Amit Misri, Sunita Maheshwari.** *Department of Pediatric Cardiology, Narayana Hrudayalaya, Bangalore.*

**BACKGROUND:** Early development of pulmonary hypertension (PAH) is unusual in atrial septal defects (ASD). Traditional teaching is that PAH develops in the 2^nd^ to 3^rd^ decade. Left superior vena cava (LSVC) is seen in 1% of general population and upto 10% of secundum ASDs. We report on a series of ASDs with early onset of PAH ie at less than 3 years of age with a high incidence of a LSVC to coronary sinus (CS).

**METHODS:** Eighty four patients (pts) were operated for secundum ASD at an age less than 3 years, 16 of whom had evidence of pulmonary hypertension (PAH). Potential etiologies for PAH were identified based on an analysis of associations.

**RESULTS:** Among these 16 pts < 3 years of age with ASD/PAH, 5 (31%) pts had LSVC which was draining in to a dilated CS into the right atrium, 2 (12%) pts had partial anomalous pulmonary venous drainage (PAPVC) with LSVC to CS, 3 (19%) pts had PAPVC, 1 (6%) pt had mitral regurgitation and in 5 (31%) cause of PAH was not known. Early onset of PAH is known in ASD and mitral regurgitation as well as in ASD and PAPVC. Thus out of 12 patients with ASD/PAH and no known cause of PAH, LSVS to CS was present in 5 (42%). Among the rest of the 68 pts <3 years with no PAH only one pt had a LSVC. Thus, in our study group of ASD/PAH 31% of pts had LSVC to CS, where as only 1.4% of pts with ASD & no PAH had a LSVC.

**CONCLUSION:** In our study 31% of patients with ASD/early PAH had a LSVC to CS as opposed to the norm of a 1-10% incidence. One could hypothesize that the left SVC and dilated CS increases the L-R shunt across the ASD and thus increases the early development of PAH. In ASD's with early PAH one must look for a LSVC-if found it could be a contributor to the PAH and early closure should be considered.

## Early outcome of neonatal blalock taussig shunt

**Pankaj Kasar, Swati Garekar, Niranjan Kumar, S Jain, S Patil, P Salvi, K Tailor, A Mhatre, Snehal Kulkarni, Z Khan, Suresh Joshi.** *Department of Pediatric Cardiology and Cardiac Surgery Wockhardt Pediatric and Congenital Heart Center, Mumbai.*

Neonatal modified Blalock Taussig shunts (mBTS) have higher perioperative and postoperative morbidity and mortality reported in India due to various reasons. We provide our institutional experience of neonatal mBT shunt.

Of 33 patients who underwent mBTS between November 2006 and August 2008, 18 were neonates (54.5%). The underlying defects were Tetralogy of Fallot/Pulmonary atresia (9), complex single ventricle (9). 6/18 patients had a right aortic arch. 14 were on PGE1 pre operatively (pre-op). The mean age at presentation was 9.6±8.9 days. The mean weight was 2.98±0.63 kg (range 2.1-3.7 kg). The midline approach was used in 17 patients. Polytetrafluoroethylene graft was used in all patients. mBTS was left sided in 5/18 (27.77%). 10/18 (55%) patients had 3.5 mm; 7/18 had 4 mm and 1 had 3mm shunt. The ductus was simultaneously ligated in 6/18 (33%). Four neonates underwent off-pump pulmonary arterial plasty concurrently.

The mean inotrope duration was 40±43hours. The mean ventilation duration was 24±0.98 hrs. Eight of 18 cases were extubated in the operative room. The mean intensive care unit stay was 3±0.97 days. The mean hospital stay was 6±1.14 days. The mean oximetry post op was 79.3%. Post op medications included sildenafil in 3, lasix in 2 and aspirin in all neonates. There was one reexploration for duct ligation for pulmonary overcirculation. Perioperative mortality was 2 (11.1%). Both babies had documented bacterial sepsis and expired of its complications. At discharge, duct was not seen in 10/16 surviving neonates. The mean duration of follow up was 6±4.66 months. There were 3 late mortalities. One had severe congenital supra-glottic stenosis, where as two had aspiration pnemonia. There were no shunt blocks. Two of 13 survivors have undergone corrective surgery to date.

In our experience, neonatal mBTS with or without ductal ligation has an acceptable early outcome.

## Minimally invasive surgery through right anterior small thoracotomy (RAST) for congenital heart defects

**Pankaj Kasar, Niranjan Kumar, Swati Garekar, Sachin Patil, S Jain, P Salvi, K Tailor, A Mhatre, Snehal Kulkarni, Z Khan, Suresh Joshi.** *Department of Pediatric Cardiology and Cardiac Surgery Wockhardt Pediatric and Congenital Heart Center, Mumbai.*

Minimally Invasive open heart surgery (MIS) is a well accepted technique with various possible approaches. We describe our method and our results.

A retrospective analysis of patients who underwent MIS of congenital heart defects at our center was done.

From November 2006 to August 2008, 26 patients qualified for the study. 11/26 were female. The mean age was 9.09±6.18 years. The mean weight was 25.65±13.7kg (Range 9-65 kg). The defects included secundum atrial septal defect (ASD) (10), sinus venosus ASD with partial anomalous pulmonary venous drainage (6) and perimembraneous ventricular septal defects (VSD) (10). 4/10 VSDs had moderate to severe pulmonary arterial hypertension. A short incision was made in the fifth intercostal space extending from the sterno-chondral edge to the right mid clavicular line. Femoro -Bicaval cannulation was done. Systemic cooling was achieved to 32° C. Myocardium was protected with antegrade cold sanguineous cardioplegia. The mean cardiopulmonary bypass time was 62.87± 15.73 minutes (Range 34-106). The mean cross clamp time was 40.52±15.4 minutes (Range 17-81). The intercostal drain was brought out through the same incision site used for bicaval cannulation. 16 patients (64%) were extubated in operating room while the rest were extubated within 6 hours post surgery. Mean duration of IV analgesic use was 22±8.1 hours. Drains were removed at a mean of 28 hrs. The average intensive care unit stay was 38.43±14.09 hours. The mean in-hospital stay was 5±0.8 days. In all cases post-operative echocardiogram suggested a complete closure of defects. There were no re-explorations. There was no early or late mortality.

Our RAST technique involves less thoracic tissue handling, minimizes trauma to structures; is less painful and more cosmetic. The ICU and ward stay is reduced. The RAST is a safe and effective alternative to a posterior/ lateral thoracotomy for correction of congenital heart defects.

## Intraoperative transesophageal echocardiography (TEE) in infants weighing less than 5 kg

**Pankaj Kasar, Swati Garekar, P Salvi, S Jain, S Patil, K Tailor, A Mhatre, Niranjan Kumar, Snehal Kulkarni, Z Khan, Suresh Joshi.** *Department of Pediatric Cardiology and Cardiac Surgery Wockhardt Pediatric and Congenital Heart Center, Mumbai.*

TEE has been demonstrated to be a safe procedure with a significant major positive impact on patient outcome. Data on the safe use and efficacy of the pediatric TEE probe in infants weighing less than 5kg is limited. We present our experience.

Between June 2007 to August 2008, there were 186 intracardiac CHD surgeries in our center out of which 68 were on infants less than 5kg. Of these TEE was planned on 46. The mean age of patients (pts) was 3.16±2.66months. Mean weight of pts was 3.76±0.93kg (2.28-5kg). 40/46 (87%) eventually underwent TEE. The TEE probe could not be inserted in 4 pts (10%) because of retrognathia. The TEE probe had to be removed prior to imaging in 2: in one case TEE probe was compressing the anomalous pulmonary venous common chamber. In the second case there were dampening of arterial trace after insertion of TEE probe in a patient with vascular ring. On 2 occasions, there was accidental extubation of the TEE probe. The mean arterial pressures increased by 5 mm of Hg after removal of TEE probe in 5 babies weighing less than 3 kg. The mean airway pressure rose by 3mm H2O in 12/40. There was no incidence of endotracheal tube dislodgement, perforation of esophagus or bleeding.

TEE findings prompted a return to CPB in 4 of 40 (10%) pts. Return to CPB occurred in 1/11 pts with ventricular septal defect, 2/17 patients with transposition, 1/5 with total anomalous pulmonary venous drainage. All post-CPB diagnoses were confirmed during reoperation.

In our experience, infants less than 5kg could safely undergo intra op TEE. The use of nasal intubation and proper case selection in infants less than 3kg contributed to the safety. The role of TEE in neonatal and infantile CHD surgery was shown to be undisputed.

## Arterial switch operation in patients with transposition of great arteries with left ventricular outflow tract obstruction

**Ritesh Sukharamwala, Sejal Shah, Kiran VS, Shekhar Rao, Chinnaswamy Reddy, Sunita Maheshwari.** *Department of Pediatric Cardiology and Pediatric Cardiac Surgery Narayana Hrudayalaya Institute of Cardiac Sciences, Bangalore.*

**INTRODUCTION:** Left ventricular outflow tract obstruction (LVOTO) is a frequent association with d-transposition of great arteries (TGA). We report our experience of different anatomical substrates for LVOTO diagnosed on echocardiography and/or per-operatively and feasibility of arterial switch operation (ASO) in those cases.

**METHODS AND RESULTS:** We retrospectively analyzed 542 patients who were operated for TGA from May 2002 to August 2008, 67 patients (12.3%) had LVOTO. Of these patients, 7 patients had intact interventricular septum (IVS) and 60 had ventricular septal defect. LVOTO was valvar in 43 patients and subvalvar in 24 patients. Subvalvar obstruction was dynamic in 5 patients and anatomical in 19 patients. Most of dynamic LVOTO were in children with intact IVS (n=4). Based on published classification of LVOTO in TGA, we have divided patients into 3 groups. Seven (36.8%) patients were of type-I (alterations in RV inflow tract including prolapse/overriding of tricuspid valve), two (10.5%) patients were of type-II (alterations in LV inflow including anomalous insertion of anterior mitral valve (MV) chordae into IVS, accessory MV tissue, fibrous ridge between MV and IVS) and ten (52.6%) patients of type-III (alterations in LVOT including posterior deviation of infundibular septum, hypertrophied septum). Three (15.7%) patients had more than one type of subvalvar LVOTO. Echocardiographic study prior to surgery had diagnosed substrate of LVOTO in most patients. ASO was feasible in 17 patients. The tissue causing LVOTO was excised without injury to conduction system and normal valve structures in all patients.

**CONCLUSION:** Our study demonstrated incidence of anatomical subvalvar LVOTO in d-TGA to be 3.5%. In certain cases of d-TGA/ LVOTO if the LVOTO is determined to be subvalvar ASO is possible instead of a Rastelli operation as was feasible in 17 of 67 patients (25%) in our study. Preoperative echocardiographic assessment of anatomical details of LVOTO, though sometimes difficult, is important as it can affect choice of surgical treatment.

## Surgical management of total anomalous pulmonary venous connection: Analysis of mortality and contributing factors

**Sudeep Verma, Ritesh Sukharamwala, Kiran VS, Shekar Rao, Chinnaswamy Reddy HM, Sunita Maheshwari.** *Deptartment of Pediatric Cardiology and Pediatric Cardiac Surgery Narayana Hrudayalaya Institute of Cardiac Sciences, Bangalore.*

**OBJECTIVES:** To evaluate outcome of total anomalous pulmonary venous connection (TAPVC) repair by a retrospective analysis of morphological and clinical profiles, with results of surgical repair.

**METHODS AND RESULTS:** Records of 414 patients (273 male, 141 female) operated for TAPVC from May 2002 to August 2008 were reviewed. Age ranged from 1 day to 40 years, with 78% being less than 1 year of age (n=323). TAPVC was categorized as supracardiac in 51% (n=212), cardiac in 31% (n=128), infracardiac in 9% (n=37) and mixed in 9% (n=37). Obstruction in pulmonary venous circuit was seen in 32% (n=131). Post-operative mortality within 4 weeks of surgery was 12% (n=49), highest being in infracardiac type (11 out of 37). The mortality was highest in younger patients, with 19% in less than 1 month old (8 out of 42). The cumulative mortality in less than one year old was 14% (46 out of 323), making younger age an important risk factor for post-operative mortality. Emergency surgery was performed in 18% (n=76) with 9% mortality (n=7). Preoperative ICU stay of > 10 days, irrespective of cause, was an important risk factor associated with postoperative mortality. Other significant risk factors for post-operative mortality were aortic cross-clamp time >100 min, CPB time >30 min and pulmonary venous obstruction.

**CONCLUSIONS:** Mortality after total anomalous pulmonary venous connection repair remains highest in young patients (likely due to more severe obstruction) and in those with infracardiac type, especially with pulmonary venous obstruction. Overall mortality of 12% as compared to western mortality statistics of 5-8% may be related to relatively delayed diagnosis and possible late referrals and higher duration of preoperative ICU stay.

## Anatomy and age of d-transposition of the great arteries vis-a-vis surgical options exercised: Analysis of 542 cases from a single center

**Ritesh Sukharamwala, Kiran VS, Sejal Shah, Shekar Rao, Chinnaswamy Reddy HM, Sunita Maheshwari.** *Deptartment of Pediatric Cardiology and Pediatric Cardiac Surgery Narayana Hrudayalaya Institute of Cardiac Sciences, Bangalore.*

**OBJECTIVE:** Arterial switch operation (ASO) is the procedure of choice for transposition of great arteries (d-TGA), typically done by 6 weeks of life. Senning procedure (SP) or Rastelli repair (RR) are considered for delayed presenters or anatomical variants. We analysed our experience with d-TGA, with respect to patient's age, anatomy and the type of surgery.

**METHODS:** Overall 658 patients were diagnosed as d-TGA from May 2002 to July 2008. Of these, 542 underwent surgery, thereby verifying echocardiographic findings. Anatomy of simple and complex d-TGA were identified and type of surgery analyzed.

**RESULTS:** The 542 patients were classified as simple (n=145) and complex (n=397). Among the simple, 50 underwent surgery within 42 days of life, with 46 undergoing ASO. SP was done due to coronary anomaly (n=4). The remaining 94 patients older than 42 days had either SP (n=77) or ASO (n=17). Three children who had ASO beyond 42 days of age expired. Thus ASO was done for infants with d-TGA intact septum older than 6 weeks in 11.7% with 82% success.

The complex subgroup had mainly ventricular septal defect (VSD) (n=257) or non-restrictive patent ductus arteriosus (PDA) (n=131). In d-TGA with VSD, 60 had left-ventricular outflow obstruction and 14 had right-ventricular outflow obstruction. Among the complex d-TGA, surgeries done were ASO (n=321), SP (n=29) and RR (n=47).

**CONCLUSIONS:** This large study from a single center in India shows that in d-TGA presenting for surgical repair, complex outnumbers simple, unlike data from the west, possibly because of early attrition due to non-referral (mainly d-TGA intact septum) versus late survival (mainly complex d-TGA). Every patient needs to undergo anatomical assessment for ASO suitability, as the left ventricle can be prepared even beyond 6 weeks, with satisfactory results. Significant coronary anomalies might be rare, but are an impediment for ASO.

## Comparative study in assessment of branch pulmonary artery size by echocardiography, angiography and CT angiography

**Yogesh Sathe, Anuradha Sridhar, Anto Sahayaraj, Shanthi C, Prem Sekar R, R Subramanian, Ravi Agarwal, KM Cherian.** *Department of Pediatric Cardiology and Pediatric Cardiac Surgery Frontier Lifeline & Dr. K.M.Cherian Heart Foundation, Chennai.*

Delineation of Pulmonary artery anatomy plays a very important role in surgical management of complex congenital heart defects. If echocardiography is unable to visualize branch pulmonary arteries, they can be visualized by either conventional angiography or noninvasive CT angiography. In this study we compared these three modalities of imaging in defining the branch pulmonary artery size.

Twenty eight patients (age 3months -34 years, weight 3.1kg – 75kg, body surface area 0.2-1.9m2) with various heart defects who had undergone echocardiogram, angiogram and CT angiogram were included in this study. Branch pulmonary artery size was measured in all three modes of imaging.

Echocardiography could not show branch pulmonary arteries in nine patients out of whom CT angiography could visualize branch pulmonary arteries in six. Conventional angiography could show branch pulmonary arteries in one more out of the remaining three patients. Statistical analysis by Fischer's test was done to compare the three modes of imaging. Fischer's test applied for comparison of conventional angiography and CT angiography revealed no statistically significant difference in their ability to visualize branch pulmonary arteries (p>0.05). We found positive correlation between these three techniques for measuring left pulmonary artery but the right pulmonary artery measurements correlated between angiography and CT angiographic measurements. CT angiography is as good as conventional angiography in visualizing and assessing size of branch pulmonary arteries and has an advantage of being noninvasive. CT angiography can be considered as a good alternative to conventional angiography if echocardiography proves inadequate.

## Utility of tele-echocardiography in pediatric cardiology as an effective diagnostic tool: A pilot project

**Ritesh Sukharamwala, Suresh PV, Bidari LH**^1^**, Sunita Maheshwari.** *Department of Pediatric Cardiology Narayana Hrudayalaya Institute of Cardiac Sciences, Bangalore and ^1^Ashwini Hospital, Bijapur, Karnataka.*

**INTRODUCTION:** Echocardiography diagnosis of congential heart disease (CHD) in India is limited by lack of trained echocardiographers with experience in the diagnosis of CHD. Tele-echocardiography has the potential to bring real-time diagnoses to pediatric facilities without in-house pediatric cardiologists. We analysed our tele-echo data to see if it made a significant difference to accurate diagnosis of CHD.

**METHODS:** At our institution tele-echocardiography is done in two ways 1) live demonstration of echo via a video camera where the cardiologist can report real time echo loops and images or 2) pre-recorded movie clips stored on a central server and retrieved by the cardiologist at a later time. From May 2006-July 2008, 218 echoes were reviewed via tele-echocardiography at 3 places, namely Bijapur, Nasik and Malaysia. We prospectively evaluated the utility of teleechocardiography in 47 children evaluated in the last 4 months.

**RESULTS:** Among the 47 patients cardiac diagnoses included VSD (n-7), ASD (n=7), PDA (n=3), TOF (n=3), TGA (n=2), TAPVC (n=1), complex CHD (n=2), myocarditis (n=1), cardiomyopathy (n=1) and normal cardiac study (n=5). After Tele-echocardiography 17 children required urgent referral for surgery, 5 children required change in treatment and 25 children did not require any changes in treatment. Out of 17 children referred, 8 were underwent successful surgery. Of the 29 children, who did not require urgent referral were asked to follow-up. Overall, the accuracy of tele-echo exceeded 95% in the study.

**CONCLUSION:** Real-time transmission of neonatal and pediatric echocardiograms has the potential to improve patient care, aid sonographer education, prevents unnecessary transports and has a positive impact on early referral of CHDs for surgery.

## Inflammatory markers are elevated in eisenmenger syndrome

**S Ramakrishnan, Amit Pandarkar, Balram Bhargava, Shyam S Kothari, Rajnish Juneja, Anita Saxena, VK Bahl.** *Department of Cardiology, All India Institute of Medical Sciences, New Delhi, India.*

**PURPOSE:** Inflammation is considered to be one of the important factors in the progression Eisenmenger syndrome (ES). Markers of systemic inflammation are not yet studied in Eisenmenger syndrome.

**METHODS:** Twenty-two consecutive symptomatic patients of ES with a mean age of 24 ± 10.6 years, were enrolled in the study. Six (27%) patients had atrial septal defect, 8 patients (36%) had ventricular septal defect and 8 patients (36%) had patent ductus arteriosus. Thirteen (59%) patients were in WHO functional class II and nine (41%) patients were in WHO class III. Inflammatory markers including hs-CRP (high sensitivity C-reactive protein), interleukin-2, interleukin-6 and interferon γ were assayed with commercially available kits using ELISA and the values were compared with 6 healthy controls.

**RESULTS:** The interim analysis of this ongoing study is presented. There is significant elevation in hs-CRP and interferon γ levels as compared to controls. The results are summarized in Table 1.

**Table 1:** Markers of systemic inflammation in Eisenmenger syndrome

Biomarker	Eisenmenger syndrome (n = 22)	Controls (n = 6)	*P* value
hs-CRP (mg/L)			
% less than 1	36	46	0.04
% 1.1 - 5	41	36	
% 5.1 - 10	14	11	
% 10	9	7	
Interferon – γ (pg/ml)	21.7 ± 12.70	8.7 ± 3.94	0.02
Interleukin – 6 (pg/ml)	12.07 ± 19.59	2 ± 1.22	0.22
Interleukin – 2(pg/ml)	19.41 ± 13.56	15 ± 12.24	0.52

**CONCLUSION:** Inflammatory markers including hs-CRP and interferon γ are significantly elevated in Eisenmenger syndrome. The causes, consequences and prognostic significance of such elevations need further evaluation.

## Nocturnal hypoxemia in patients with eisenmenger syndrome

**S Ramakrishnan, Rajnish Juneja, Shyam S Kothari, Anita Saxena.** *Department of Cardiology, All India Institute of Medical Sciences, New Delhi, India.*

**INTRODUCTION:** Sleep studies have shown significant nocturnal hypoxemia in patients with severe pulmonary disease and primary pulmonary hypertension. Sleep studies in Eisenmenger syndrome (ES) have not been reported.

**METHODS:** The study included 16 patients with ES and 8 patients with cyanotic congenital heart disease (CCHD) with decreased pulmonary blood flow (PBF) as controls. All the patients underwent an overnight comprehensive polysomnogram study. An oxygen drop was defined as any fall in SpO_2_ greater than 5% lasting for at least 9 seconds.

**RESULTS:** The patients and controls showed significant nocturnal hypoxemia in the absence of apnea and hypopnea. In ES patients, an increased ODI (oxygen drop index) was significantly related to lower SpO_2_ at baseline (p = 0.04) and after 6 min walk (p = 0.001), sleep disturbances (p = 0.04), increase in REM sleep, and a lower DLCO value (p = 0.03). Patients with ODI > 10 had significantly higher hematocrit (54 ± 3.9% Vs 47.7 ± 3.2%, p < 0.05) and hemoglobin levels (16.9 ± 1.2% Vs 14.9 ± 1.4%, p = 0.03) than those with ODI ≤ 10.

**CONCLUSION:** Eisenmenger syndrome patients have significant nocturnal hypoxemia unrelated to hypopnea and apnea. Nocturnal desaturation occurred more frequently during REM sleep, and in patients with greater hemoglobin and hematocrit values. These findings may have important therapeutic implications.

## Tissue doppler imaging in evaluating transposition of great arteries (TGA)

**S Ramakrishnan, Indra Kuladhipati, Sandeep Seth, Rajnish Juneja, Shyam S Kothari, Anita Saxena.** *Department of Cardiology, All India Institute of Medical Sciences, New Delhi, India.*

**BACKGROUND:** Tissue Doppler and Strain rate imaging are relatively load independent. We studied the usefulness of Tissue Doppler and Strain rate imaging in detecting temporal changes in regional and global function of left and right ventricle in transposition of great arteries (TGA).

**METHODS:** Standard echocardiography including Doppler studies was performed with 5 or 10 MHz probe (GE Vivid 7 dimension Machine). The images were analyzed offline by a customized software package (Echo Pac PC,GE Vivid Ultrasound). Intra and inter-observer variability was assessed to be within standard limits. The correlation with surgical outcome will be presented.

**RESULTS:** A total of 35 patients (aged 3- 123 days) were included in this study. Twelve LV and 2 segments of RV in each patients were analyzed for peak systolic velocity, systolic strain rate and strain. There were no significant intergroup differences in of any of these parameters in patients with or without VSD, or in the groups with regressed or prepared LV. Paradoxically, peak systolic velocity and peak strain rates were statistically significantly higher in patients older than 28 days.

**CONCLUSION:** Conventionally classified as regressed LV do not show reduced tissue velocities and strain rates. Peak systolic velocity and peak strain are higher in patients more than 28 days. These findings suggest that possibly intrinsic contractility of the LV myocardium is preserved in a majority of patients with TGA.

## Cruciate fenestration in the ventricular septal defect patch – medium term followup of high risk patients

**Anuradha Sridhar, Nithya lakshmi, Anto Sahayaraj, Prem Sekar R, Raghavan Subramanyan, Ravi Agarwal, KM Cherian.** *Department of Pediatric Cardiology and Pediatric Cardiac Surgery Frontier Life line Hospital & Dr.K.M.Cherian Heart foundation, Chennai.*

**ABSTRACT:** Closure of Ventricular septal defect (VSD) is associated with high risk of suprasystemic Right ventricular (RV) pressure and RV failure in complex cyanotic defects with dimunitive pulmonary arteries and in shunt lesions with severe pulmonary artery hypertension. Large fenestrations cause excessive left to right shunt after the RV pressure declines and need closure. To avoid this, a single 3 mm cruciate fenestration was made in the VSD patch in high risk patients.

**METHODS AND RESULTS:** Between January 2004 and december 2007, seventy four patients had fenestrated patch closure of VSD. Among these, retrospective analysis of Forty one Indian patients who are on regular followup was done. They were divided in to four groups:

**GROUP 1-** Appropriate closure of fenestration with normalization of RV pressure (31.7%)

**GROUP 2-** Premature closure of fenestration before normalization of RV pressure (12%)

**GROUP 3-** Delayed closure of fenestration (open even after normalization of RV pressure) (17.7%)

**GROUP 4-** Appropriately functioning fenestration in the presence of persistent high RV pressure (39%)

One patient died among those in group 2 with premature closure. Among group 3 patients,though there was delay in closure, the size of the fenestration had decreased during followup and none of them required fenestration closure.

**CONCLUSION:** To conclude, cruciate fenestration in the VSD patch in high risk patients allows effective decompression of RV in the immediate postoperative period with little need for reintervention later.

## Mid-term results of the modified ross/konno procedure in neonates and infants

**Katsuhide Maeda, Rachel Rizal, Sam Suleman, Olaf Reinhartz, Daniel J Murphy, Frank L Hanley, V Mohan Reddy.** *Department of Pediatric Cardiac Surgery Lucile Packard Children's Hospital Stanford University, USA*

**OBJECTIVE:** Congenital aortic stenosis in infants is sometimes complicated by hypoplastic annulus or diffuse subaortic stenosis. In this subset of patients the mid to long term results of Ross-Konno procedure with respect to the fate of autograft valve and reoperations were evaluated.

**METHODS:** Between 1994 and 2007, 20 patients under 1 year of age underwent the Ross-Konno procedure. The diagnoses were aortic stenosis with or without subaortic stenosis (n=12), Shone complex (n=6), interrupted aortic arch with subaortic stenosis (n=1). On average, 1.2 previous balloon dilation interventions had been performed per patient. The aortic root was replaced with a pulmonary autograft valve and the LVOT was enlarged with a right ventricular infundibular free wall muscular extension harvested in continuity with the autograft.

**RESULTS:** Age at surgery ranged 1 - 236 days (median 29 days). The follow ranged from 1 to 159 months (median 61 month). There were no deaths. By echocardiography, 5 patients developed trace to mild neo-aortic regurgitation, and two developed moderate regurgitation, requiring aortic valve repair at the time of allograft conduit replacement. None required valve replacement. The growth of the autograft paralleled somatic growth and the right and left ventricular functioned returned to and remained normal. In addition, 15 reoperations & reinterventions were performed, including 10 pulmonary homograft replacements, 1 percutaneous pulmonary xenograft valve implantation, 1 mitral valve replacement, 1 coarctation repair, 1 right coronary artery patch augmentation and 1 pacemaker implantation.

**CONCLUSION:** The pulmonary autograft demonstrated good durability without development of aortic stenosis or severe insufficiency. This study shows that modified Ross-Konno procedure may be an ideal choice for complex LVOT stenosis with significant annular and subannular hypoplasia in neonates and infants.

## Effects of adenosine for pulmonary hypertension crisis – experiment of twelve pigs

**Kunihiko Tonari.** *Pediatric Cardiovascular Surgery, Kyorin University School of Medicine, Pediatric Cardiovascular Surgery, Tokyo, Japan.*

**BACKGROUND:** The mechanism for onset of postoperative pulmonary hypertensive crisis in congenital heart disease patients accompanying pulmonary hypertension (PH) is still unclear, and in many cases may greatly complicate treatment. The purpose of this study was to examine the pharmacological effects of adenosine on pulmonary hypertension.

**METHODS:** Twelve pigs with induced experimental pulmonary hypertension (age, about 2 months, mean weight 10.9 kg) were studied under general anesthesia. Experimental pulmonary hypertension was induced by hypoxic inhalation with FiO2=0.1. Data was collected for mean systemic artery pressure (MAP), mean pulmonary artery pressure (MPAP), central venous pressure (CVP), left atrial pressure (LAP), and cardiac output. Cardiac index, MAP, MPAP, CVP, LAP, pulmonary vascular resistance (PVR), systemic vascular resistance (SVR), PVR/SVR ratio, and MPAP/MAP ratio were determined before, during, and after central venous infusion of adenosine (20 to 80μg/kg/min) for 30 minutes. Statistical analysis was performed using ANOVA and Tukey's post hoc analysis, and statistical significance was defined as a P-value of less than 0.05.

**RESULTS:** Effective adenosine concentration was 40μg/kg/min (11 out of 12 cases). Concerning the effects of adenosine on experimental acute PH in pigs, MPAP significantly decreased from 26.9 mmHg to 17.3 mmHg (p < 0.01), and PVR significantly decreased from 4.2 Wood units (W.u.) to 1.9±1.1 W.u. (p < 0.01). No change in systemic circulation during adenosine infusion was observed.

**CONCLUSION:** This study showed that adenosine selectively decreased PAP and PVR without decreasing systemic blood pressure and SVR. The results of this study suggest that adenosine at low concentrations may be clinically effective for the treatment of perioperative and postoperative pulmonary hypertension for infants and young children.

## Psychosocial and neurodevelopment outcome in children and adolescents undergoing surgery for congenital heart disease

**SM Reddy, A Nehra, B Airan, AK Bisoi, A Saxena, SS Kothari, P Venugopal.** *Department of Cardiothoracic Surgery, All India Institute of Medical Sciences, New Delhi, India.*

**BACKGROUND:** As advances in the medical and surgical management improves survival in patients with congenital heart disease, increasing knowledge about neurodevelopmental and psychosocial outcomes and the factors that affect them will provide strategies to optimize long-term outcome in this high-risk population.

**OBJECTIVE:** To evaluate the neurodevelopmental and psychosocial outcome in children with various types of congenital heart defects (both cyanotic & acyanotic) who underwent corrective surgical procedures from birth till 15 years of age. This investigation is part of an institutional effort to examine the neurodevelopment of children who underwent corrective surgical procedures for congenital heart defects.

**PATIENTS AND METHODS:** We performed a battery of neuropsychological tests on a sample of 70 children with congenital heart defects (35 patients each in cyanotic group and acyanotic group) between 2006 and 2008 at 6 months and 1 year following surgical correction.

**RESULTS:** The overall psychosocial and Neurodevelopmental outcome was found to be satisfactory in both the groups when assessed by standard psychosocial instruments for respective age. There was no significant difference in the overall outcome between the cyanotic and acyanotic group. The subgroup of patients who performed below average had their surgical corrective procedure at more than two years of age and psychological counseling was offered to all the patients.

**CONCLUSION:** Further regular long term follow up is required to identify the specific psychosocial problems if any in these patients. More research regarding age at surgical repair and the psychosocial outcome is needed. There is an important need for further studies of quality of life in patients with different types of congenital heart diseases.

## Primary arterial switch beyond 3 weeks of age: what is feasible without ECLS?

**G Kumar, R Gupta, M Tomar, SK Kaushal, PU Iyer, S Radhakrishnan, S Shrivastava, KS Iyer.** *Department of Pediatric Cardiology and Department of Pediatric Cardiac Surgery Escorts Heart Institute and Research Centre, New Delhi.*

**BACKGROUND:** Primary arterial switch beyond 3 weeks of age and subsequently beyond 2 months of age has been shown to be a feasible option in infants with d transposition of great arteries, intact ventricular septum (dTGA.IVS) with possible need for mechanical support as a rescue strategy (Foran et al, Kang et al ). In Dec 2005, we carefully considered the possibility of primary arterial switch in such infants as opposed to our earlier practice of atrial switch ( Senning operation ) or the rapid two stage switch (parental preference).

**OBJECTIVE:** To evaluate the outcome of primary arterial switch in infants with dTGA.IVS beyond 3 weeks of age without mechanical support as a rescue strategy.

**DESIGN:** A prospective pilot observational study.

**SETTING, PATIENTS AND METHODS:** 46 infants underwent primary arterial switch for d TGA.IVS during this period. 22 were ≥ 3 weeks of age ( late Group) - median age 55 days, 22-149 days. Preop ECHO in these 22 infants showed varying LV mass (30-43gm/m^2^) and LV morphology ranging from favorable to biventricular dysfunction. Post repair ECHO revealed varying degrees of LV dysfunction in 20/22. Perioperative low cardiac output was managed premptively using multiple, simple, inexpensive conventional strategies. Differences in primary (mortality) and secondary ( measures of morbidity) outcome between the 2 groups (late and early) were analyzed using Fisher's extract test, chi square, Mann-Whitney U and student t test as appropriate.

**RESULTS:** In-hospital mortality was similar in both groups (late: 1/22 and early: 2/24, p=0.6). Median ventilatory requirement ( late -115 hours versus early -94.5 hours), length of stay (late -11.5 days versus early -12 days) and inotrope score were similar in both groups respectively. There were no differences in the incidence of renal failure, acute lung injury and sepsis in the two groups.

**CONCLUSION:** Preliminary observations suggest that primary arterial switch in infants ≥ 3 weeks of age using simple, inexpensive ICU strategies is feasible. These observations need to be validated in larger numbers of older infants to decide “how old is safe?”.

## Clinical screening for congenital heart disease immediately after birth: A prospective study

**Gayathri S, Balu Vaidyanathan, Sinimol TM, Sundaram KR, Warrier KRR**^1^**, Anand KM**^1^**, R Krishna Kumar.** *Department of Pediatric Cardiology Amrita Institute of medical Sciences and ^1^Laksmi Hospital, Kochi.*

**BACKGROUND:** A large proportion of neonates born with congenital heart disease (CHD) are missed at birth in Indian hospitals.

**OBJECTIVE:** To develop a clinical strategy for detection of congenital heart disease (CHD) in the newborn through a combination of clinical signs and pulse oxymetry that best predicts the presence of CHD.

**METHODS:** All consecutive newborns born in a secondary level hospital between June 2006 and June 2008 were prospectively screened for CHD 48 hours after birth. The on-site pediatrician performed clinical screening for CHD using a pre-designed format of 10 clinical signs. Pulse oxymeter saturations (right upper and lower extremity) were recorded by a nurse. Echocardiography was performed on site in all newborns. A 6-week clinical follow-up evaluation was also performed.

**RESULTS AND ANALYSIS:** Of the 4190 babies screened, 325 had CHD (overall prevalence: 7.7%). Nine (0.21%) had major CHD (3 cyanotic & 6 acyanotic), two of whom (one ALCAPA and one large VSD) were missed during the initial evaluation and were detected on follow-up; four of these had a normal clinical evaluation and only one had low resting oxygen saturation. Univariate analysis of predictors of CHD included murmur, central cyanosis, precordial pulsation, and respiratory rate >60/minute. On multivariate analysis, murmur (OR=5.8; 95% C.I 3.44 -9.76) and respiratory rate >60/min (OR=2.54; 95% C.I 1.0 – 6.47) were associated with presence of CHD. The overall low sensitivity of the clinical signs (7.2% for murmur) perhaps was due to small number of patients with significant CHD. Pulse oximetry was not found to be a significant predictor of CHD possibly due to technical and human factors.

**CONCLUSIONS:** Clinical evaluation immediately after birth has a very low sensitivity for predicting newborns with CHD. Presence of murmur in a newborn warrants a referral for echocardiography. A 6-week repeat clinical evaluation is recommended to ensure that major CHD does not go undetected.

## Chromosome 22q11 microdeletion in patients with conotruncal malformations: Prevalence and phenotypic correlates

**Mahesh K, Alka Anil Kumar, Sheela Nampoothiri, MV Thampi, KR Sundaram, R Krishna Kumar.** *Department of Pediatric Cardiology, Pediatric Genetics and Human Cytogenetics Amrita Institute of Medical Sciences, Kochi, Kerala.*

**BACKGROUND:** There is little data on prevalence of 22q11 microdeletion syndromes among children with conotruncal malformations in Indians. Presence of this deletion has significant impact on the perioperative management and long-term outcome.

**OBJECTIVES:** To determine prevalence and identify the best phenotypic correlates of chromosome 22q11 microdeletion among patients with conotruncal malformations.

**METHODS:** This was a prospective, hospital-based, observational study conducted at a tertiary care center on consecutive children (≤ 2 years) with conotruncal cardiovascular anomalies that were identified and classified by pediatric cardiologist using echocardiography. A pediatric geneticist independently evaluated patients for dysmorphic features and phenotype. Karyotyping, and Fluorescence *In Situ* Hybridization (FISH) using the critical region probe for 22q11, was then performed.

**RESULTS:** FISH was positive for microdeletion 22q11 in 26 (20.8%) of 125 consecutive patients with conotruncal malformations studied (TOF: 14%, TOF with pulmonary atresia: 27.1%, DORV: 23.1%, conoventricular VSD: 21%, truncus arteriosus: 50% and type B interrupted aortic arch: 60%). On univariate analysis a statistically significant (p <0.05) association was found between 22q11 microdeletion and dysplastic or flared pinna, bulbous nasal tip, micrognathia, thin long fingers, high arched palate, hypoplastic ala nasii, microstomia, prominent hooked nose, microcephaly and hypocalcemia. Multivariate logistic regression analysis showed that thin long fingers (OR: 13.24, p = 0.001), bulbous nasal tip (OR: 7.35, p = 0.011), facial weakness/asymmetry (OR: 22.83, p = 0.029), high arched palate (OR: 4.65, p = 0.025), and dysplastic flared pinnae (OR: 4.19, p = 0.04) were in 90.4% agreement with FISH positivity for 22q11 microdeletion.

**CONCLUSIONS:** A significant proportion of Indian Children with conotruncal malformations have 22q11 microdeletion. Identification of the combination of typical phenotypic features described may help in early clinical prediction of 22q11 microdeletion. This in turn may allow improved perioperative care pending the results of FISH.

## Decline in arterial PO_2_ after exercise can help stratify patients with unrestrictive atrial septal defect and pulmonary hypertension

**Srinivas L, Anil Singhi, KR Sundaram, R Krishna Kumar.** *Department of Pediatric Cardiology Amrita Institute of Medical Sciences, Kochi, Kerala.*

**BACKGROUND:** It is frequently challenging to determine operability in patients with large atrial septal defects (ASD) and pulmonary arterial hypertension (PAH) using conventional means.

**OBJECTIVES:** To examine the utility of decline in arterial partial pressure of oxygen (PaO_2_) after exercise as a marker of pulmonary vascular obstructive disease (PVOD) in patients with ASD with PAH.

**METHODS:** A symptom limited treadmill exercise was performed in three groups of patients: Healthy volunteers (n = 6), large ASD with no PAH (n = 5) and 14 patients with ASD and PAH (oxygen saturation ≥ 90%, predicted PA systolic pressure of ≥ 50 mm Hg by Doppler echocardiography in absence of additional lesions or lung disease). Patients with advanced PVOD (sats < 90%) were excluded. A radial artery cannula was inserted before exercise and arterial blood gas samples were drawn before and as soon as the test was terminated (peak exercise). A decline in PaO2 ≥ 10 mmHg after exercise was considered significant. The patients with ASD and PAH underwent cardiac catheterization and detailed hemodynamic data sets were obtained on room air, oxygen and, a mixture of oxygen and nitric oxide (30-40 ppm).

**RESULTS:** None of the controls (healthy or ASD and no PAH) had a significant fall in PO_2_. The data obtained from patients with ASD and PAH (n = 14, age: 33.6±8.6 yrs) is shown in the table 1.

**Table 1:** Results

Parameter		≥ 10mm drop in PaO_2_ after exercise (n=8)	< 10mmHg drop in PaO_2_ after exercise (n=6)	*P* value
Basal PaO_2_		77.3 ± 14.2	80 ± 12.1	NS
PVRI > 7	Basal	8	1	0.001
wood	On O_2_	6	0	0.008
units.m^2^	On NO	5	0	0.013
PVRI < 7	Basal	0	5	0.001
wood	On O_2_	2	5	0.008
units.m^2^	On NO	1	5	0.013
Mean PA	Basal	60 ± 12.7	40 ± 10.8	0.01
pressure	Change with O_2_	-0.63 ± 2.06	-7.0 ± 4.1	0.022
(mm Hg)	Change with NO	-3.86 ± 4.4	-9.2 ± 5.8	NS

NS: Non significant

**CONCLUSIONS:** A decline in PaO_2_ following exercise appears to predict a high PVRI (basal, post O_2_ and post NO) in patients with ASD and PAH. This test appears promising for assessment of operability in borderline situations.

## Pulse oximetry as a screening tool for detecting congenital heart defects in the british midlands – An interim analysis

**Abhay Bhoyar**^1,2^**, John G C Wright**^2^**, Andrew Ewer**^1,3^. *^1^University of Birmingham, Edgbaston, B15 2TT, ^2^Cardiology Department, Birmingham Children's NHS Foundation Trust, Steelhouse Lane, B4 6NH, ^3^Neonatal Unit,*Birmingham Women's *NHS Foundation Trust, Metchley Park Road, Edgbaston* , *B15 2TG,*Birmingham, UK.

**OBJECTIVE:** To determine the accuracy of Pulse oximetry (PO) for detecting critical and clinically significant CHD in newborn.

**STUDY DESIGN:** This population based prospective multicenter delayed cross sectional study was conducted in 6 the hospitals in the British Midlands. Asymptomatic newborns ≥35 weeks of gestation had arterial oxygen saturations measured within 24 hours of birth. A cut off of <95% in either limb or a difference of ≥3% between the limb readings was considered as abnormal. If PO was low and the clinical examination was unremarkable, the PO was repeated 1-2 hours later for a definitive definition of abnormality. Echocardiogram and interrogation of various databases were used as gold standards to confirm CHD.

**RESULTS:** Of the 17500 deliveries in the 6 hospitals from February 2008 to September 2008, 13733 (78.5%) babies have been recruited and screened using PO. The mean birth weight was 3.31 Kg and the male to female ratio was 1.04. The number of parents declining the consent for the study was relatively higher amongst the Asian and the Black populations, 20% and 25% respectively Vs White population, <10% ( p<0.0001). Of the infants screened, 140 (1.01%) failed the test. Twenty eight (20%) had CHDs, 18 (12%) of them were critical CHDs, who required either surgery or catheter intervention within the 4 weeks of birth. Of the 28 CHDs, 22 (15.7%) were true positives, whereas 6 (4%) were false negatives. Pulmonary or other disorders were present in 50 (35.5%) neonates. Of the 28 CHDs, 9 were antenatally diagnosed, 8 out of 9 were true positives and one was false negative. The prevalence of critical and clinically significant CHD in our population was 2 per 1000. For identifying critical and clinically significant CHDs, the PO screening had sensitivity rate of 78.5% (95% CI, 58.5 to 90.9), specificity rate of 99.1% (95% CI, 98.9 – 99.2) and false positive rate of 0.8%.

**CONCLUSION:** Pulse oximetry screening promotes early detection of CHDs. The sensitivity rate for detecting critical CHDs is high and the false positive rate is low.

## Clinical evaluation of the new amplatzer duct occluder II (ADOII)

**Chetan Mehta, Vinay Bhole**^1^**, Oliver Stumper**^1^**, Paul Miller**^1^**, John Wright**^1^**, Joseph DeGiovanni**^1^**.** *Department of Pediatric Cardiology Royal Manchester and Alder Hey Children's Hospital, ^1^Birmingham Children's Hospital, United Kingdom.*

**INTRODUCTION:** The success rate of transcatheter closure of PDA has improved with evolving newer generation of devices. A newer generation of ductal device is now available from Amplatzer with a new design. We present our initial evaluation of this device ADO II.

**OBJECTIVES:** To evaluate the new Amplatzer ADO II device for safety, immediate and late closure rate, complications and device behaviour during catheterization

**METHODS:** Device selection performed as per manufacturer's recommendations. Patient demographics, device sizes, occlusion rate, behaviour and complications documented. All patients had angiograms performed before and after release of the device. Echocardiography evaluation was done either on the evening or the day after the procedure.

**RESULTS:** Twenty- three patients had transcatheter PDA occlusion with the ADO II device. The median age was 22 months (7 months – 68 years); median weight was 11.7 kg (4.5 – 77.3 kg). The mean arterial duct diameter was 3 mm (2 – 4.4 mm). The approach for device deployment was arterial in 11 and venous in 12 patients. Angiography after device release showed complete closure in 14 and trivial flow through the device in 9 patients. The median procedure time was 43mins (15 – 82mins) and the fluoroscopy time 6mins (2.2 – 26.5mins). No residual shunts were seen on colour Doppler on the following day. There were no complications.

**CONCLUSIONS:** The new ADO II device is safe and effective for PDA's with various shapes and lengths with diameters up to 5.5 mm. The articulation, high early closure rate, arterial/venous approach options and small diameter delivery catheter are all beneficial features of this new device.

## Need of cardiac catheterization in immediate post operative period with congenital heart diseases (CHD)

**M Tomar, S Radhakrishnan, KS Iyer, S Shrivastava.** *Department of Pediatric Cardiology and Department of Pediatric Cardiac Surgery Escorts Heart Institute & Research Centre, New Delhi.*

Retrospective study of all patients who underwent surgery for CHD over a 7-year period (June 2001 to July 2008).

**MATERIAL AND METHODS:** Out of 3624 patients who underwent surgery for CHD at our Institute 18 patients (0.5%) (12 males, 6 females), age group 22 days-23 years underwent cardiac catheterization in immediate post-operative period (2^nd^ to 16^th^ postoperative day). Details given in table 1.

**Table 1:** Details of Cases

No	Cardiac cath Diagnostic/intervention	Age group	Surgery	Post-op day	Results and remarks
1	Diagnostic for persistent LCO	2 m(n-1)	Truncus type 1 repair	6	Severe stenosis of both branch PAs at conduit–PA junction(kinked), successfully reoperated Moderate sized Residual VSD
		23y(n-1)	TOF total correction with TAP	15	Moderate sized Residual VSD RV pressure 70% systemic RVEDP 15 mmHg, No PS Reoperated but died on 25th POD
2.	Coiling of MAPCs n=8	22 d –8m	TOF n=4	2-10	All were successfully weaned from ventilator
			Complete TGA with intact IVS or small VSD n=2		
			Complete TGA with large VSD, mild PS, PAH n=1		
			Scimitar syndrome n=1		
3.	Balloon valvotomy n=2	22-23 days	Coa in post arterial switch operation n=1	6 & 10	Good results in both
			BPV post closed surgical valvotomy (n=1)		
4.	VSD device closure, n=1	1 year	Post VSD surgical closure Additional muscular VSD	9th	Died after 2 days cause sepsis
5.	Coiling of venovenous collateral, n=1	1 year	Post bidirectional Glenn shunt, persistent low SpO2	14nd	Large venovenous collateral which was successfully coiled and child weaned off ventilator
6.	Balloon dilatation of BT shunt, n=1	2 months	Right modified BT shunt On echo-blocked BT shunt	4th	Successful balloon dilatation
7.	Echo guided Balloon atrial septostomies, n=3	1-5 months	Tricuspid atresia,VSD,PS. Post BT shunt	2-7th	Successful septostomy. 5 months old baby died due to persistent low output stage

**RESULTS:** All patients required intervention (2 were reoperated, 13 underwent successful catheter intervention). All but two were successfully weaned off from ventilation. Three patients died due to persistent low output state could be due to delay in reintervention (intervention was done after 6 days).

**CONCLUSION:**

1. One should consider for cardiac catheterization in post operative period if patient is not stable and condition can not be explained by echocardiographic findings. Best outcomes follow expedient catheterization with definitive management.
2. Constant vigil of post operative patients in the ICU can diagnose significant residual defects which may be amenable to non surgical intervention leading to successful weaning of otherwise unstable patients.

## Catheter closure of atrial septal defects with deficient inferior vena cava rim under transesophageal echo guidance

**KS Remadevi, Edwin Francis, Raman Krishna Kumar.** *Division of Pediatric Cardiology, Amrita Institute of Medical Sciences and Research Centre, Kochi*.

**OBJECTIVES:** To describe the case selection, imaging considerations, technique and results of catheter closure of atrial septal defects (ASD) with deficient inferior vena cava (IVC) rim.

**BACKGROUND:** Transcatheter closure has become standard treatment for most secundum ASDs. Defects with deficient IVC rim continue to be challenging to image and close in the catheterization laboratory.

**METHODS:** Records of 28 patients with deficient IVC rim (< 5mm), who underwent catheter closure (April 2007- August 2008) were reviewed. This represented 14% of the total numbers of patienst undergoing ASD device closure in the study period. General anesthesia and transesophageal echo (TEE) guidance were used in all. Amplatzer Septal Occluder (ASO, AGA Medical, Golden Valley, MN) was used in 13 and HeartR ASD occluder (Lifetech Scientific Co., Ltd, Shenzen, China) in 15 patients. The IVC rim was imaged at 70 to 90 degrees with retroflexion of the TEE probe, in addition to the conventional views. Devices 1-4 mm > maximal ASD size was selected. Deployment was accomplished either from the left atrium, left upper or from the right pulmonary veins.

**RESULTS:** The median age was 6.75 (3-33) years and median weight was 20 (9-81) Kg. The defects measured 16-41 mm and 18-42 mm septal occluders were used. The median fluoroscopic time was 13.4 (3.7-65) minutes. Six patients had trivial residual flows at IVC margin. Complications were device embolization in four (retrieved in three and two underwent successful closure with same or larger device), transient atrial flutter in two and, transient Mobitz type I second-degree heart block in two patients. All patients are well at follow up (median: 5; range: 1-16 months).

**CONCLUSIONS:** Transcatheter closure of ASDs with deficient IVC rim is feasible in selected patients under TEE guidance. The modified retroflexed view allows adequate imaging of IVC rim through TEE.

## Use of amplatzer vascular plug for complex congenital interventions

**S Ramakrishnan, SS Kothari, R Juneja, A Saxena.** *Department of Cardiology, All India Institute of Medical Sciences, New Delhi, India*.

**OBJECTIVES & METHODS:** Amplatzer vascular plug (AVP) is an underutilized item in the armamentarium of interventional cardiologists, though it is less thrombogenic than the duct occluder. We describe 9 cases of unusual interventions using the AVP.

**RESULTS:** The patient age ranged from 2 – 20 years. The diagnosis included coronary aterio-venous fistula (3 cases), baffle leak following Fontan operation (1 case), Coil embolization of AP collaterals (2 plugs), LPA aneurysm (1 case) and post operative BT shunt (Through arterial approach 1 case and through Glenn circuit 1 case). In 3 of the cases, delivery of duct/septal occluder failed due to inability to adequately advance the delivery sheath or the occluder. 4mm – 12 mm AVP were used. In all the cases, the delivery of AVP was relatively unproblematic as it could be advanced over a 5F or 6F right coronary guiding catheter. The procedure was successful in all the cases. Complete occlusion of shunt could be achieved with AVP alone, despite concerns over less thrombogenicity.

**CONCLUSION:** In selected cases, Amplatzer vascular plug offers an alternative innovative solution for the interventionists. The better profile and manoeuvrability was useful in all the cases.

## Feasibility and midterm outcome of transcatheter closure of small subaortic VSD with aortic cusp prolapse

**NA Radzi**^1^**, M Alwi, H Samion, H Latif, G Kandavello.** *Department of Pediatric Cardiology, National Heart Institute, Kuala Lumpur, Malaysia*

**OBJECTIVE:** Small subaortic ventricular septal defect is not a benign lesion and aortic regurgitation is recognized as a late complication. This study looked at the feasibility and medium term outcome of transcatheter closure of small subaortic ventricular septal defect with aortic cusp prolapse +/- aortic regurgitation (AR) as an alternative to surgical closure.

**METHODS:** Between July 2004 to February 2006, prospectively collected data of 65 patients who underwent TEE and cardiac catheterization were analysed. 52 had perimembraneous and 13 had doubly-committed subarterial type VSD. Patients with weight more than 10 kg, haemodynamically insignificant shunt and presence of aortic valve prolapse +/- trivial or mild aortic regurgitation were initially included. Standard technique was performed to close the VSD using AMVOD. The patients were followed up for a minimum of 2 years.

**RESULTS:** The mean age was 249 months (range 14-300). All of them had cusp prolapse with the degree of prolapse trivial in 50 (77%), mild in 10 (15%), moderate in 4 (6%) and severe in 1 (2%). 11 were excluded on TEE. 43 out of 50 (86%) had successful closure. 7 (14%) developed severe aortic regurgitation necessitating device retrieval. 4 cases were abandoned due to procedural difficulties. 36 (84%) patients showed no worsening of AR. Only 7 (16%) patients had worsening of AR, 3 from nil to trivial, 3 from trivial to mild and one progressed to severe AR requiring aortic valve replacement. 6 patients had transient arrhythmia and one developed new RBBB.

**CONCLUSION:** Transcatheter closure of small subaortic ventricular septal defect with mild aortic cusp prolapse + aortic regurgitation is feasible, safe and offers an attractive alternative to conventional surgical closure.

## Hypertensive VSD – device closure can be a good approach: Our experience

**T Madan, N Patel, J Shah, D Patel, S Shah, BM Thakkar.** *Department of Pediatric Cardiology U. N. Mehta Institute of Cardiology & Research Centre, Ahmedabad, Gujarat*.

**BACKGROUND:** The percutaneous closure of perimembranous ventricular septal defect (VSD) is accepted as a feasible option nowadays. The hypertensive VSD can also be closed percutaneously without much complication. Some times hybrid approach like perventricular closure of defect can be done which to avoid the risk of cardiopulmonary bypass and its complications in very small children.

**OBJECTIVE:** Retrospective study to analyse the safety and efficacy of Block Aid VSD occluder (Perimembranous & Muscular) for treatment of VSD with severe pulmonary artery hypertension (PAH).

**PATIENTS & DESIGN OF STUDY:** We enrolled 17 patients with age 4 months to 14 years. There height was 92 ± 30 cms. & weight was 11.6 ± 6.87 kg. Four of them had perimembranous VSD and thirteen had muscular VSD. The resting saturation was 98% and right ventricular systolic pressure (RVSP) was 75.05 ± 10.74 mmHg. 14 patients underwent percutaneous closure and 3 underwent per ventricular closure of VSD. We used 7 to 9 Fr. delivery sheaths depending on size of devices. VSD sizing was done after left ventricular (LV) angiogram in left anterior oblique (LAO) 60°, cranial 20° and LAO 45°, cranial 25° angulated views. All patients of Perimembranous VSD were also looked for absence of aortic regurgitation (AR) & adequacy of aortic rim for device closure. All patients were followed at 7 days, one month, three months and then at six months interval.

**RESULTS:** The mean size of VSD was 9.95 ± 3.03 mm on echocardiography and 9.22 ± 1.5 mm on angiography. Defect was successfully closed in all the patients with trivial residual shunt in 5 patients & complete closure in the rest of patients. The RVSP prior to closure was 75.05 ± 10.74 mmHg and after closure was 57.47 ± 7.68 mmHg. The pulmonary artery (PA) systolic pressure was 61.5 ± 3.12 mm Hg before the procedure and came to 49.23 ± 5.72 mm Hg after the procedure.. Fluoroscopy time was 25.01 ± 11.15 min. Two patients developed conduction disturbances. On follow up all had normal sinus rhythm. Three patients had blood loss & required Blood Transfusion. One patient expired post procedure due to intracerebral haemorrhage.

**CONCLUSION:** Block Aid VSD occluder can be considered for closing large VSDs with severe PAH. In small children due to non-accessibility of arterial access with large sheaths, perventricular closure is a feasible option for closure of VSD. Further studies are required to document is efficacy, safety & long-term results in large number of patients.

## Catheter closure of ductus arteriosus in symptomatic low birth weight preterm infants

**Srinivas L, Mahesh K, Edwin Francis, Balu Vaidyanathan, R Krishna Kumar.** *Department of Pediatric Cardiology Amrita Institute of Medical Sciences, Kochi*.

**BACKGROUND:** Medical or surgical treatment of symptomatic patent ductus arteriosus (PDA) in preterm infants is well-accepted. Transcatheter treatment of these PDA in very small infants is technically challenging.

**OBJECTIVES:** To describe our institutional experience with transcatheter coil closure of PDA in symptomatic low birth weight preterm infants.

**METHODS:** Case selection for the transcatheter procedure was determined by the patient's weight, PDA size, size of ampulla vs. the anticipated coil size required for complete closure (determined through echocardiography). PDA occlusion was achieved with multiple coils delivered with assistance of a 3F bioptome. Arterial access and catheter manipulation within the cardiac chambers were avoided whenever feasible.

**RESULTS:** 8 preterm infants underwent coil occlusion. Their gestational age ranged form 27 to 32 weeks (28.7 +/- 1.9 weeks). Their birth weights ranged from 700 to 1700grams (1144 +/-348 grams) and they weighed between 900 to 1800grams (1205+/- 285 grams) at the time of procedure. All of them had symptoms of heart failure, that included tachypnea and poor weight gain. Three patients were mechanically ventilated before the procedure. Size of the PDAs ranged between 2mm and 3.5mm. Complete occlusion of the duct was achieved in 7 of them on the table and one had a tiny residual flow that resolved by next 24 hours. No major procedure or access related complications were encountered. 4 patients were discharged by 72 hrs, one was discharged on day 10 and the other 2 required prolonged ventilation (34 and 28days) due to associated pulmonary pathology.

**CONCLUSION:** It is technically feasible to successfully perform transcatheter coil closure of PDA in carefully selected symptomatic preterm infants.

## Experience with transcatheter closure of secundum type atrial septal defects in children with a self expanding nitinol double disc device: A single centre study over a period of 2 years

Samarasinghe Duminda*, Narenthiran Sinnathurai, Fernando Nimali, Santharaj Wijeyasingam, Perera Shehan, Irugalbandara Sunethra, Goonetilleke Mangal, Weerasuriya Dimuthu, Morawakkorala Ruwan, Senarath Upul^1^**.** *Department of Paediatric Cardiology, Lady Ridgeway Children's Hospital, ^1^Department of Community medicine, University of Colombo, Colombo, Sri Lanka*.

**AIM:** To evaluate safety and efficacy of transcatheter closure of Secundum Atrial Septal Defects (ASD) in a newly established unit in first two years.

**METHODS:** A single centre retrospective review of 264 consecutive patients with a significant ASD who were considered for transcatheter closure with a self expanding Nitinol double disc device were included in the study. Four patients were excluded due to anatomical and physiological issues. Inability to implant a device according to manufacturer's recommendations was taken as a failure.

**RESULTS:** In our study population mean age was 7.6 (± 2.8) years and mean body weight was 20.26 (± 7.43) kg. Defect size ranged from 6 to 27mm (mean 14.2 ± 4 mm). Out of 260 patients, a device was successfully implanted in 214 (82.3%) whereas it was not successful in 46 (17.7%). Balloon sizing was done only in 12 (4.2%) and was not done in 254 (95.8%). Amplatzer Septal Occluder (ASO) was used in 230, Amplatzer multi-fenestrated Septal Occluder in 1 and Blockaid Septal Occluder (BSO) in 29. Mean fluoroscopy time was 12.7 (± 10.6) mins. Failure rate was 25% (n = 25) in first 100 patients, while it has decreased significantly to 13.1% (n = 21) in the subsequent 160 (P=0.015).Multivariate analysis revealed that likelihood of failure was higher in patients with suboptimal margins (OR=35.93; P=0.000), device larger than 20mm in size (OR=6.36; P=0.005) and procedures done during early phase (OR=6.80; P=0.001). There was no significant association between failure and age of patient or device type (ASO or BSO). Ten patients had minor complications and four patients had major complications including two device embolization and one death due to cardiac perforation.

**CONCLUSION:** Efficacy of Transcatheter closure of ASD in a newly established unit has improved over the period of time and complications are comparable to published data in other centres.

## Stenting of native coarctation of aorta in children using adult iliac stents

**AN Patnaik, D Seshagirirao.** *Department of Cardiology Nizam's Institute of Medical Sciences, Hyderabad*.

Balloon angioplasty followed by stent is now an accepted strategy for the treatment of native coarctation of aorta in older children and adolescents world over. For the last 5 years we used self expanding nitinol stents (primarily recommended for iliac angioplasty in adults) in this condition. There were 32 patients (24 males; mean age 10.2± 1.43 years; youngest 6 year old). Successful procedure is defined as reduction of gradient to less than 20 mmHg or increase in the ratio of the diameter of the coarctation area to the diameter of the descending aorta to at least 0.8. In 32 patients 32 stents were used with overall success rate of 31/32 (96.8 %). The peak systolic pressure gradient (mean (SD)) decreased from 48.8 (23.5) to 2.2 (1.86) mm Hg (p<0.05). The diameter of the stenotic lesion increased from 5.6 (1.6) mm to 12.5 (2.6) (p <0.05). In one patient 2 stents were used because the first stent migrated downwards leaving the lesion partially uncovered. In the only unsuccessful case the lesion was 12 mm long, tubular and did not yield even at 16atm pressure. This case was sent for elective surgery at a later date. There were no deaths or cerebrovascular events. Two cases had femoral artery access related problems (hematoma-1, loss of pulse-1). Twenty five of the 31 successful cases were on regular clinical and echocardiographic follow-up. On mean follow-up of 1.25 years one had recoartation and successful balloon dilation was done. Another case with recoartation and persistent hypertension underwent elective surgical repair. There were no aortic aneurysms in any case. Stent implantation using this readily available low cost adult peripheral stent gave gratifying acute and early term results in treatment of coarctation of aorta in older children and adolescents.

## A study of percutaneous closure of ventricular septal defect by duct occluder

**N Patel, H Vasavda, T Madan, M Kharache, H Gandhi, BM Thakkar.** *U. N. Mehta Institute of Cardiology & Research Centre, Ahmedabad, Gujarat*.

**BACKGROUND:** Majority of patients with muscular VSDs amenable to device closure can be closed with conventional muscular VSD occluder (MVSDO). However in certain cases either the defect location and shapes or the device design dose not appear very appropriate particularly which are located more apically and having narrow and long right ventricular (RV) exit. The Block-aid Duct Occluder (BDO) can be a good and suitable device in such cases.

**OBJECTIVE:** Retrospective analysis of safety and efficacy of Block-aid duct occluder for the treatment of apical and mid muscular conical VSD

**PATIENT AND DESIGN:** Six patients aged 4 to 9 years with height of 115.8 ± 10.76 cm (range 99-130 cm) and weight of 16.83 ± 3.76 kg (range 14-25 kg) with apical or mid muscular VSDs underwent transcatheter closure using BDO. The device consists of nitinol wire mesh that is shaped in to a cylindrical plug with a collar and with a polyester fabric inserted in it. Selection of delivery system and duct occluder size was according to LV Angiographic delineation of defects.

**RESULTS:** Transthoracic echocardiography showed VSD size of 4.71 ± 1.56 (LV), 3.27 ± 0.90 (RV). The RVSP was 41.66 ± 9.46. The angiographic VSD diameter was narrowest 3.19 ± 0.85 and largest 5.04 ± 2.56. A 6 to 9 French sheath was used to deliver the device. BDO of sizes 6x4, 8x6, and 10x8 were used according to the VSD sizes. Successful device delivery and complete closure occurred in all patients. Fluoroscopy time was 14.13 ± 8.36 min (range 8.54-32.12). No complications occurred.

**CONCLUSIONS:** BDO is an important adjunct for closure of muscular VSDs and further studies are required to document its efficacy, safety, and long term results in a larger number of patients.

## Twin procedures in pediatric cath lab – our experience

**Amit Misri, Pankaj Bajpai, Suresh PV, Sunita Maheshwari.** *Department of Pediatric Cardiology, Narayana Hrudayalaya, Bangalore*.

**BACK GROUND:** From relatively simple procedure like patent ductus arteriosus (PDA) closure to comparatively difficult ones like ventricular septal defect (VSD) closure, pediatric cardiac interventions have come a long way. With ever evolving technology plus increasing experience, two transcatheter interventions in the same patient (twin procedures) can be now performed with good results. We report our experience of twin procedures from 2005-2006.

**METHODS:** All patients whose age was less than 18 years and had underwent more than one cath intervention for two different diagnosis at the same sitting were considered. Patients who had undergone two procedures either for two different disease or same disease but at different sittings were excluded.

**RESULTS:** Nine patients whose age ranged from 6 months to 18 years with mean of 6.5 years underwent twin procedures. Out of these 5 (55%) had PDA and atrial septal defect (ASD) closure, 2 (22%) had ASD closure and balloon pulmonary valvuloplasty (BPV), 1 (11%) patient had BPV and balloon aortic valvuloplasty (BAV) and 1 (11%) patient had BAV and balloon coarctoplasty. All the procedures were successful with no immediate or short term complications.

**CONCLUSION:** Twin procedures, whenever possible, are an effective alternative to surgery. Short post procedural ICU care and ability to do them off bypass are advantages. However cost implications and the amount of metal inserted into body may be issues for discussion.

## Interventional therapy of pulmonary arteriovenous malformation using PDA or ASD occluder

**Wu Wenhui, Xu Liang, Xu Zhong–ying, Jiang Shi–liang, Huang Lian–jun, Zhao Shi–hua, Zheng Hong, Ling Jian, Zhang Ge–jun.** *Department of Radiology, Cardiovascular lnstitute & FuWai Hospital, Chinese Academy of Medical Sciences, Beijing*.

**OBJECTIVE:** To evaluate the efficacy of transcatheter occlusion of Pulmonary artery-venous malformation (PAVM) by using PDA or ASD occluder.

**MATERIALS AND METHODS:** The patients included 4 females and one Male with age range of 7-21years. Angiography showed diffuse capillary pulmonary arterio-venous malformation, multiple saccular and single saccular in 2,2,and 1 cases respectively. Pulmonary artery-venous malformation was occluded by transcatheter technique in all patients using PDA or ASD occluder.

**RESULTS:** Technical success was achieved in all patients. Hypoxemia symptoms were relieved. The arterial mean oxygen saturation was increased from 75.2% to 92.7%.

**CONCLUSION:** Interventional therapy by using the PDA or ASD occluder is an effective treatment for pulmonary-arteriovenous malformation which have the huge saccular and the large feeding artery.

## Transcatheter occlusion of ruptured sinus of valsalva aneurysm with amplatzer duct occluder

**Zhao Shihua, Yan Chaowu, Zhu Xianyang, Xu Naixun, Jiang Shiliang, Xu Zhongying, Wang Cheng, Wu Wenhui, Hu Haibo, Li Shiguo, Ye Zhankai, Wang Hao.** *Department of Radiology, Cardiovascular Institute and Fuwai Hospital, Chinese Academy of Medical Sciences and Peking Union Medical College, Beijing, China*.

**BACKGROUND:** Ruptured sinus of Valsalva aneurysm (RSVA) can be associated with ventricular septal defects or isolated lesions. Percutaneous transcatheter closure of RSVA has been an alternative strategy to surgery.

**METHODS:** From January 2000 to May 2006, 10 patients (4 males, 6 females) aged from 7 years to 69 years (mean ages 37+/-18.8 years) were involved in the present report. The diagnosis of RSVA was made based on a combination of several imaging modalities. Of them, 9 patients were identified as congenital cause and one did as acquired RSVA. Two-dimensional and color Doppler echocardiography revealed the rupture of right coronary sinus into right ventricle in 5 cases and into right atrium in 3 cases, while non-coronary sinus ruptured into right atrium in 2 cases. Aortogram showed that the estimated size of the defect was 6.2+/-2.3 mm (2-10 mm). After the establishment of the arterio-venous wire loop, Amplatzer duct occluder (ADO) was deployed by antegrade venous approach in all patients.

**RESULTS:** ADO with 1-3mm larger than the defect was used. All defects were successfully occluded without any complications. On the follow-up, echocardiography showed neither residual shunt nor aortic regurgitation, and there was also no device embolization, infective endocarditis in any of the patients.

**CONCLUSIONS:** Transcatheter closure is a feasible and effective alternative for both congenital and acquired RSVA. However, long-term follow-up is mandatory.

